# Retrospective slice prescription compensation improves coronary cross-sectional area measurement by MRI

**DOI:** 10.1186/1532-429X-13-S1-P236

**Published:** 2011-02-02

**Authors:** Travis Smith, Krishna Nayak

**Affiliations:** 1 University of Southern California, Los Angeles, CA, USA

## Objective

To determine if vessel orientation can be estimated retrospectively, and if this information improves measurements of coronary luminal area.

## Background

**Figure 1:**
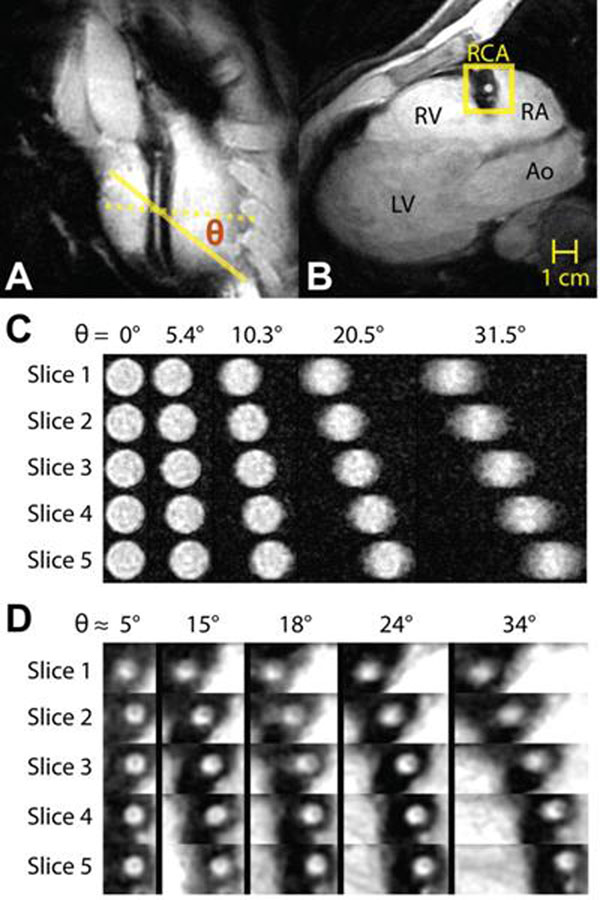
(a) Long axis view of a linear RCA segment with slice prescription angle θ. (b) Cross-sectional image of the RCA. (c) Cross-sectional images from the cylindrical vessel phantom. (d) Cross-sectional images around the RCA for each estimated slice angle θ

Measurements of coronary cross-sectional area are utilized in vasomotor tone and endothelial function studies, which typically employ breath-held, 2-D multi-slice imaging protocols wherein slices are prescribed orthogonally to a linear vessel segment (Fig 1a) [1],[2]. An ovoid template is then manually fitted to the transverse vessel images (Fig 1b) to measure cross-sectional area. Projection through the slice leads to dependencies between the measured area and the prescription angle (between the slice and the vessel--ideally 0°). This adds bias and reduces repeatability, which is problematic when detecting subtle dilations [3].

## Methods

Cardiac-gated, breath-held spiral coronary angiography with spectral-spatial excitation was performed using a GE Signa 3T scanner (5 mm slice thickness, 16 interleaves, 5 slices). Transverse views of a cylindrical vessel phantom (diameter=5 mm, field-of-view=5 cm, resolution=0.25 mm) and a linear segment of the right coronary artery (RCA) in a healthy volunteer (diameter=~3.5 mm, field-of-view=22 cm, resolution=0.7 mm) were acquired, along with field maps, for gridding reconstruction and off-resonance deblurring [4]. We applied a segmented cylindrical model and estimated the prescription angle for each slice from the vessel displacement through the two neighboring slices. The measured cross-sectional areas were then geometrically compensated to correct any apparent ellipticity from non-orthogonal prescriptions. This process was repeated with several prescription angles ranging from 0° to approximately 30°.

## Results

**Figure 2:**
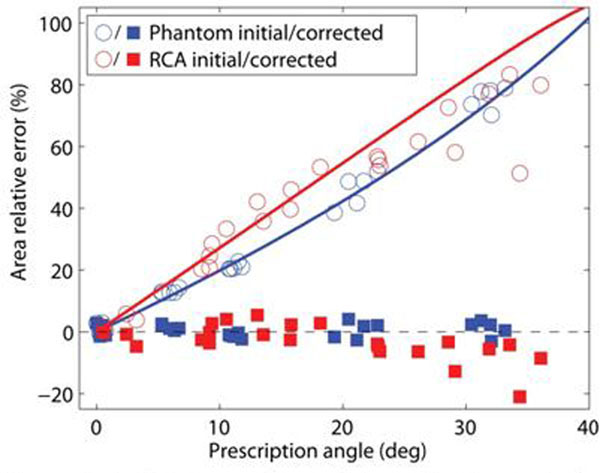
Relative error for each image in Figure 1c,d for both initial (circle) and corrected (square) area measurements. The solid lines indicate the theoretical performance based on our geometric model. Relative error = 100x(measured-true)/true.

Non-orthogonal prescription angles lead to slice-dependent elliptical projections of the cylindrical phantom (Fig 1c) and the RCA (Fig 1d). Ellipticity is also generated by changes in the local RCA shape, as seen in the 5°, slice 1 image. Our approach reduced area measurement error at every prescription angle (Fig 2). The approach is sensitive to angle estimation accuracy, and tends to underestimate area as seen in the high-angle *in vivo* results. A higher-order model of the vessel may improve estimation performance. Nonetheless, the error performance after correction was equivalent to acquiring with much smaller prescription angles (Tables 1,2). The median *in vivo* improvement in the sensitivity reduction was 16-fold.

**Table 1:** Performance in the vessel phantom

True angle (deg)	Estimated angle (deg)	Area relative error % (initial)	Area relative error % (corrected)	Error-equivalent angle (deg)	Prescription sensitivity reduction factor
0	0.3 ± 0.1	0.7 ± 2.0	0.08 ± 1.8	0.04	0
5.4	5.6 ± 0.6	12.7 ± 0.3	1.7 ± 1.1	0.9	6
10.3	11.2 ± 0.3	21.1 ± 1.3	-1.0 ± 0.6	0.5	21
20.5	20.3 ± 0.9	43.0 ± 5.1	-0.07 ± 3.6	0.04	512
31.5	31.9 ± 1.4	74.3 ± 4.4	-0.06 ± 2.7	0.03	1050

The error-equivalent angle is the prescription angle corresponding to the mean area error after correction (based on the solid lines in Fig. 2). The prescription sensitivity reduction factor is the ratio of the actual prescription angle to the error-equivalent angle.

**Table 2:** Performance in the RCA

True angle (deg)	Estimated angle (deg)	Area relative error % (initial)	Area relative error % (corrected)	Error-equivalent angle (deg)	Prescription sensitivity reduction factor
	4.5 ± 5.3	13.5 ± 17.4	1.0 ± 2.6	0.4	11
	15.1 ± 7.1	40.2 ± 14.6	-0.4 ± 5.9	0.2	75
	17.8 ± 10.4	41.5 ± 19.3	-4.4 ± 7.7	1.6	11
	23.5 ± 8.1	57.8 ± 18.7	-4.0 ± 1.5	1.5	16
	33.5 ± 7.4	80.4 ± 17.6	-5.3 ± 1.1	2.0	17

## Conclusions

Cross-sectional lumen area can be retrospectively compensated for non-orthogonally prescribed slices, thereby reducing the sensitivity to slice prescription and breath-hold repeatability. This approach should reduce operator dependence and shorten localization time.
